# Data on blueberry peroxidase kinetic characterization and stability towards thermal and high pressure processing

**DOI:** 10.1016/j.dib.2017.05.044

**Published:** 2017-05-26

**Authors:** Netsanet Shiferaw Terefe, Antoine Delon, Cornelis Versteeg

**Affiliations:** CSIRO Agriculture and Food, Australia

**Keywords:** Blueberry, Peroxidase, Enzyme kinetics, Thermal processing, High pressure processing

## Abstract

The data presented in this article are related to a research article entitled ‘Thermal and high pressure inactivation kinetics of blueberry peroxidase’ (Terefe et al., 2017) [1]. In this article, we report original data on the activity of partially purified blueberry peroxidase at different concentrations of hydrogen peroxide and phenlylenediamine as substrates and the effects of thermal and high pressure processing on the activity of the enzyme. Data on the stability of the enzyme during thermal (at temperatures ranging from 40 to 80 °C) and combined thermal-high pressure processing (100–690 MPa, 30–90 °C) are included in this report. The data are presented in this format in order to facilitate comparison with data from other researchers and allow statistical analyses and modeling by others in the field.

**Specifications table**

**Table:** 

Subject area	*Food science, Biochemistry, Food engineering, Food processing*
More specific subject area	*Food biochemistry*
Type of data	*Table, figures*
How data was acquired	*Spectrophotometry (UV-1700 Pharmaspec, Shimadzu, Japan), High pressure processing (35L-600 sterilization machine, Avure Technologies, USA and #U111 high pressure kinetic unit, Unipress, Warsaw, PL), thermal processing*
Data format	*Raw and analyzed data*
Experimental factors	***Substrate concentration:** constant excess concentration of hydrogen peroxide (0.44 M) and phenylenediamine concentration from 0 to 0.5 M, constant excess concentration of phenylenediamine (0.092 M) and hydrogen peroxide concentration from 0 to 1.5 M.* ***Temperature**: Temperatures between 20 and 100 °C* ***Pressure**: pressures between 100 and 690 MPa* ***Processing time**: 0–100 min*
Experimental features	*The experimental design included kinetic investigations and response surface experimental design methodology*
Data source location	*Highbush blueberry grown in Victoria, Australia*
Data accessibility	*The data are available with this article*

**Value of the data**

• The data on the activity of blueberry peroxidase at varying concentration of the reactants phenylenediamine and hydrogen peroxide and after thermal and high pressure processing gives an insight into the kinetic and stability properties of the enzyme to other researchers interested in processing blueberries or using peroxidase as a biocatalyst.
• The data set can be used by researchers interested in developing new statistical and kinetic models for characterizing enzyme activity and the combined effects of high pressure and heat on the activity of enzymes.
• The data can be used for comparison to other studies on plant peroxidases.

## 1 Data

The data reported in this paper is related to a research article entitled ׳High pressure and thermal inactivation kinetics of blubbery peroxidase׳ (Terefe et al., 2017)[1]

**Fig. 1:**
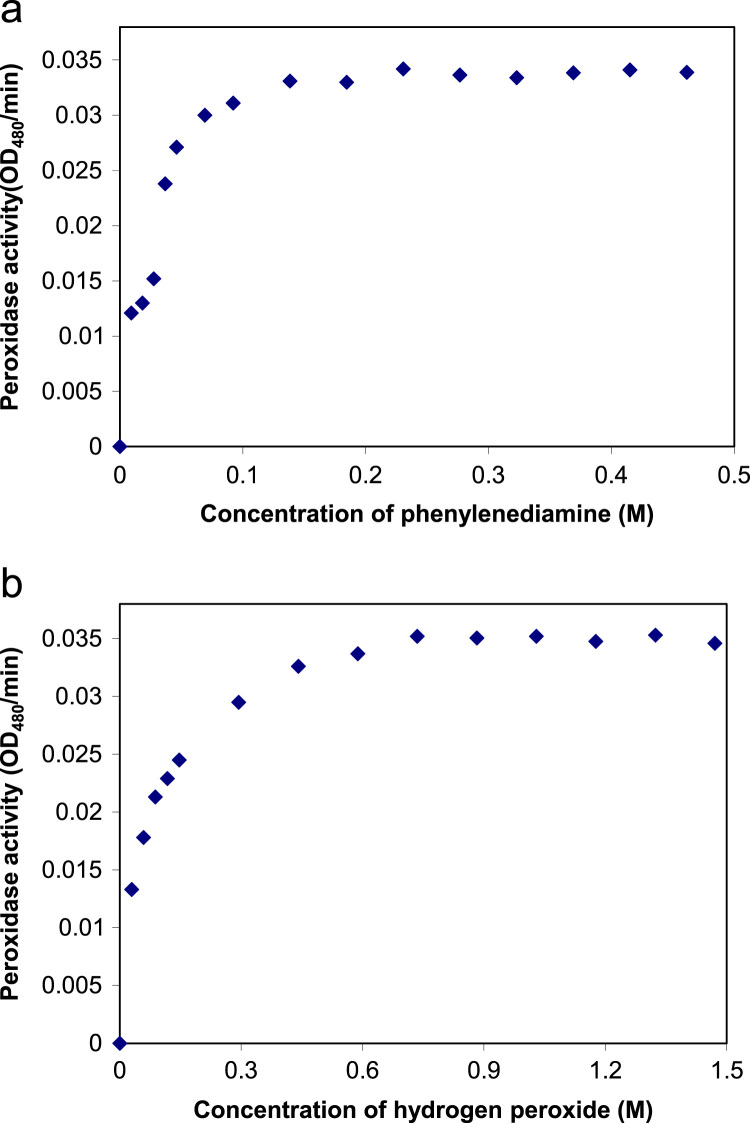
a. Data on blueberry peroxidase activity as a function of phenylenediamine concentration (McIlvaine buffer (pH=5.5), 25 °C) at constant excess concentration of hydrogen peroxide (0.44 M). b. Data on peroxidase activity as a function of the concentration of hydrogen peroxide (McIlvaine buffer (pH=5.5), 25 °C) at constant excess concentration of phenylendiamine (0.092 M).

**Fig. 2:**
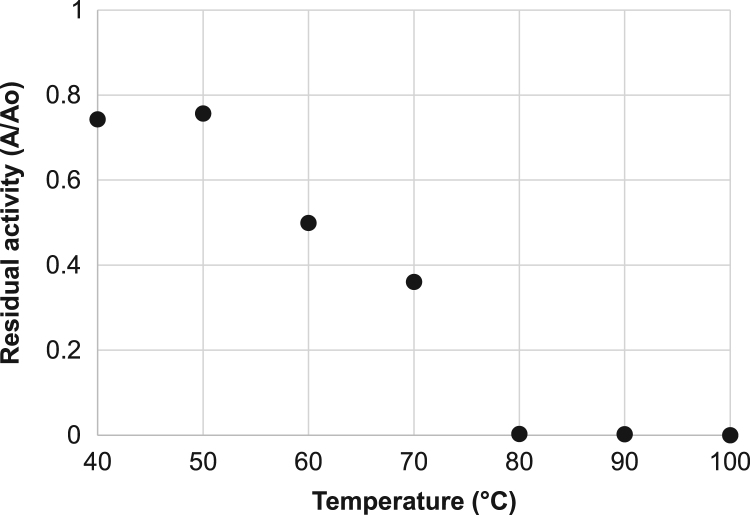
Data on the effects of thermal treatment (20–100 °C for 10 min) on the activity of blueberry peroxidase in McIlvaine buffer (pH 3.6).

**Fig. 3:**
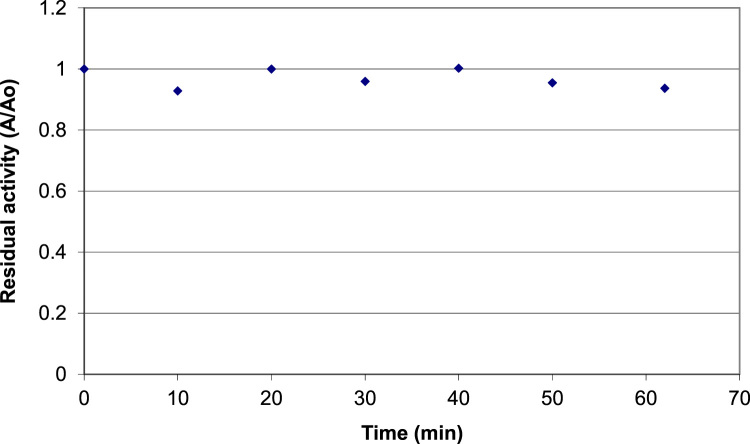
Data on the effect of high pressure treatment at 400 MPa and 30 °C on the activity of blueberry peroxidase in McIlvaine buffer (pH 3.6).

**Fig. 4:**
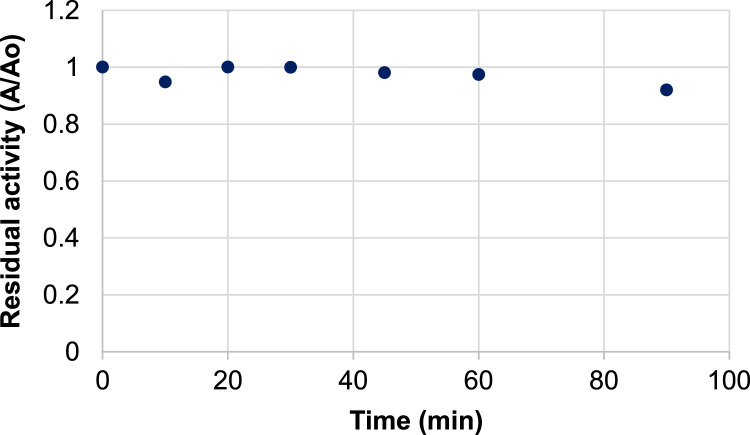
Data on the effect of high pressure treatment at 500 MPa and 40 °C on the residual activity of blueberry peroxidase in McIlvaine buffer (pH 3.6).

It includes data on the activity of blueberry peroxidase 1) at different concentrations of phenylenediamine (0 to 0.5 M) and constant excess concentration of hydrogen peroxide (0.44 M) (Figs. 1(a) and 2) at different concentrations of hydrogen peroxide (0–1.5 M) and constant excess concentration of phenylenediamine (0.092 M) (Fig. 1b). Data on the effects of thermal processing on the activity of blueberry peroxidase are presented which show the residual activity of the enzyme after 10 min incubation at temperatures ranging from 20 to 100 °C (Fig. 2). Data on the effect of combined high pressure-temperature processing (temperature 30–90 °C and pressure 100–690 MPa, hold time, 15 min) on the activity of blueberry peroxidase are presented in Table 1. Data on the effect of combined high pressure-thermal processing (at 400 MPa and 30 °C and 500 MPa, 40 °C) for up to 100 minutes on the activity of the enzyme are given in Fig. 3, Fig. 4.

**Table 1:** Original experimental data on the effects of combined high pressure–temperature processing for 15 min on the activity of blueberry POD in McIlvaine buffer (pH 3.6). The percentage residual activities ((*A*/*A*_o_) x100) are presented where *A*_o_ represent the activity of the enzymes in the untreated samples and *A* represents the activity of the enzyme after processing.

**Run**	**Temperature (°C)** *(X*_*1*_*)*	**Pressure (MPa)** *(X*_*2*_*)*	**POD (% residual activity)** (*Y*_*1*_)
1	90	690	0.5
2	70	100	70.2
3	70	690	65.5
4	50	100	82.6
5	70	690	66.9
6	50	395	78.1
7	70	395	142.5
8	90	100	0.33
9	90	100	0.36
10	70	395	140.7
11	70	395	125.9
12	70	395	102.1
13	70	395	130.4
14	70	395	156.4
15	50	690	72.05
16	70	395	162.9
17	70	100	73.04
18	90	395	81.01
19	50	100	80.7
20	90	395	65.8
21	70	395	176.8
22	50	690	72.8
23	70	395	180
24	90	690	135.4
25	50	395	78.3
26	70	395	142.5
	**30**	**100**	**92.3**
	**30**	**100**	**90.3**
	**30**	**395**	**104.4**
	**30**	**395**	**103.9**
	**30**	**690**	**78.6**
	**30**	**690**	**75.7**

## 2 Experimental design, materials and methods

Peroxidase was extracted from homogenized highbush blueberry and partially purified as described in Terefe et al. [2]. In order to determine the kinetic parameters of the enzyme with respect to its two substrates, phenylenediamine and hydrogen peroxide, the activity of the enzyme was evaluated, at constant excess concentration of hydrogen peroxide (0.44 M) varying the concentration of phenylenediamine and constant excess concentration of phenylenediamine (0.092 M) varying the concentrations of hydrogen peroxide.

The data on the effects of thermal processing on peroxidase activity was obtained by incubating enzyme samples in 100 μl capillary tubes in a thermostated water bath maintained at temperatures ranging from 40 °C to 100 °C for 10 min. After each treatment, samples were cooled in ice water and the residual enzyme activity was assayed in accordance with the method of Terefe et al. [3]. All experiments were conducted in duplicates.

The data on the effects of combined high pressure-thermal processing on the activity of blueberry peroxidase was acquired by conducting experiments using response surface experimental design with three levels of the experimental variables and their 9 combinations (Table 1). The experiments were conducted in a 35 L high pressure vessel (35L-600 sterilization machine, Avure Technologies, USA) The activity of the enzyme was assayed prior to and after processing for 10 minutes at the different pressure-temperature combinations.

The kinetic data on the effects of combined high pressure-thermal processing was obtained from experiments at different pressure and temperature conducted in a bench-scale high pressure kinetic unit (#U111, Unipress, Warsaw, PL) at 400 and 500 MPa and 30 and 40 °C under isothermal-isobaric conditions. The activity of the enzyme was assayed after pressure-temperature equilibration (time 0) and following treatments for different processing times. All experiments were conducted in duplicates.

## References

[1] Terefe N.S., Delon A., Versteeg C. (2017). Thermal and high pressure inactivation kinetics of blueberry peroxidase. Food Chem..

[2] Terefe N.S., Delon A., Versteeg C. (2015). Blueberry polyphenol oxidase: characterization and the kinetics of thermal and high pressure activation and inactivation. Food Chem..

[3] Terefe N.S., Yang Y.H., Knoerzer K., Buckow R., Versteeg C. (2010). High pressure and thermal inactivation kinetics of polyphenol oxidase and peroxidase in strawberry puree. Innov. Food Sci. Emerg. Technol..

